# The Mediating Role of Inner Strength in the Relationship Between Biological Factors and Depressive Symptoms

**DOI:** 10.3390/nursrep15100348

**Published:** 2025-09-25

**Authors:** Jia Jiao, Rewadee Jenraumjit, Shirley Worland, Saifon Bunyachatakul, Bijing He, Tinakon Wongpakaran

**Affiliations:** 1 Mental Health Program, Multidisciplinary and Interdisciplinary School, Chiang Mai University, Chiang Mai 50200, Thailand; jia_jiao@cmu.ac.th (J.J.); bijing_he@163.com (B.H.); 2 Department of Pharmaceutical Care, Faculty of Pharmacy, Chiang Mai University, Chiang Mai 50200, Thailand; 3 Faculty of Health Sciences, University of New England, Armidale, NSW 2351, Australia; sworland@une.edu.au; 4 Department of Occupational Therapy, Faculty of Associated Medical Sciences, Chiang Mai University, Chiang Mai 50200, Thailand; saifon.bun@cmu.ac.th; 5 Longquan Secondary Vocational School, Longquan 323700, China; 6 Department of Psychiatry, Faculty of Medicine, Chiang Mai University, Chiang Mai 50200, Thailand

**Keywords:** mental health, depressive symptoms, biological factors, inner strength, working women, China, Nursing

## Abstract

**Background:** Depression is a significant public health concern, with working mothers at greater risk due to combined biological and psychosocial stressors. Recent evidence suggests that inner strength may play a mediating role in the link between biological risks and depression. **Objectives:** The primary objective was to determine whether inner strength mediates the relationship between biological risk factors (hormonal fluctuations, smoking, alcohol use, family psychiatric history, and physical diseases) and depressive symptoms among Chinese working mothers. A secondary objective was to assess the prevalence of depressive symptoms in this population, measured with the OI-Depression subscale (Outcome Inventory-21). **Methods:** A cross-sectional online survey was conducted with 330 Chinese working mothers aged 30–45 years, using validated instruments to measure depression, inner strength, and parental stress. Mediation analysis evaluated the indirect effect of inner strength, while covariate-adjusted regression analyses explored associated factors. **Results:** Biological risk factors showed a significant direct effect (β = 0.584, *p* < 0.001) and an indirect effect through inner strength (indirect effect = 0.623, 95% CI [0.294, 0.962]. The Sobel test indicated that the indirect effect of biological risk factors on depressive symptoms through inner strength was statistically significant (z = 3.67, *p* < 0.001). The prevalence of clinically significant depressive symptoms was 38.2%. **Conclusions:** Biological factors significantly contribute to depressive symptoms, but inner strength partially mediates this relationship, suggesting that interventions to enhance inner strength may help reduce depression risk in working mothers. Further research should investigate strategies to build inner resilience in this population.

## 1. Introduction

Based on the Global Burden of Diseases 2019 Study, the global number of individuals living with depression climbed from 172 million in 1990 to 258 million in 2017, demonstrating a 49.86% increase [1]. WHO reports that around 54 million Chinese citizens suffer from depression [2]. Enhancing the mental health status of the Chinese population through public health policies could yield significant benefits worldwide.

Working mothers refer to women with children who are engaged in full-time or part-time employment while simultaneously bearing the primary responsibilities of caregiving and household tasks [3]. In Chinese society, which is deeply influenced by Confucian cultural values, women have traditionally been expected to fulfill family-centered roles [4]. However, with the rising cost of urban living, particularly in housing, education, and healthcare, an increasing number of households rely on dual incomes from both parents to maintain their standard of living [5]. According to a 2020 survey conducted in China, approximately 35% of mothers were employed in full-time jobs, and an additional 8.4% held part-time jobs or operated a secondary business [6]. Work-family conflict is particularly pronounced for women in the middle years of life [7]. A previous study found that work-family conflict significantly increases depressive symptoms and employment-related guilt among working mothers aged 25–47, highlighting that mothers in this age bracket often face heightened role strain due to overlapping professional and caregiving responsibilities [8]. Another study emphasized that feelings of overwhelm, emotional distance from children, and role overload were strongly associated with poor mental health outcomes, particularly among middle-aged working mothers [9]. The mental health of working mothers represents an urgent public health concern, especially in rapidly developing societies like China. Addressing the mental health needs of working mothers is critical to promoting social equity, fostering sustainable workforce participation, and strengthening community resilience [10].

Women have an inherent biological vulnerability to depressive symptoms when encountering stress related to other psychological and environmental factors. Women may have a higher sensitivity to stress-related neurochemical changes, which can affect mood stability [11]. The biological factor includes hormonal fluctuation that can have an impact on women’s mental health. Up to 30% of pregnant women experience anxiety, but it is often underdiagnosed [12]. Rapid postpartum drops in estrogen and progesterone are linked to postpartum depression (affecting 25% of new mothers) [13]. Women undergoing the menopausal transition often experience anxiety and depression symptoms because hormonal changes may influence brain regions like the hypothalamus and hippocampus, affecting neurotransmitter systems such as serotonin and GABA, leading to mood disorders [14]. Notably, another biological risk factor that renders one more likely to develop depressive symptoms and other psychiatric conditions was highest among those with a family history involving two generations affected by depression [15]. Furthermore, physical diseases that are considered secondary biological factors, such as hypertension, dyslipidemia, and diabetes, are associated with depression, particularly pronounced among females [16]. Moreover, bio-behavioral factors related to lifestyle, such as alcohol consumption and cigarette abuse, are found to be risk factors for depressive symptoms. Analysis of data from older adults in Ireland revealed that current smokers were significantly more likely to experience symptoms of anxiety and depression compared to non-smokers, indicating a strong association between tobacco use and mental health challenges in later life [17]. These findings are consistent with previous research conducted among Australian adolescents [18]. In addition, individuals with current alcohol dependence exhibited a greater likelihood of persistent depressive disorders over a two-year follow-up period [19].

While risk factors for women have been extensively investigated, protective factors, especially modifiable or psychological factors, are limited. Studies demonstrated that inner strength, an individual’s capacity to actively confront and cope with adversities and challenges [20], could be one of them. In the mentioned study, patients with a high level of inner strength are more capable of mobilizing external resources to manage disease progression and treatment, thereby enhancing adherence to therapy and maintaining a better quality of life despite diseases [21]. Another study examining inner strength identified ten positive behavioral traits: truthfulness, perseverance, wisdom, generosity, morality, mindfulness or meditation, patience and endurance, equanimity, determination, and loving-kindness, which found that, among Chinese salespersons, inner strength would have a negative correlation with anxiety [22]. According to national health statistics, there were approximately 219,574 community nurses in China in 2020 [23]. A study has demonstrated that even brief sessions of loving-kindness meditation can modulate brain wave activity associated with emotional regulation, particularly in beta and gamma frequencies within the amygdala and hippocampus, which may contribute to reductions in depressive symptoms [24]. The integration of psychological nursing with mindfulness-based stress reduction (MBSR) significantly alleviates depressive symptoms, enhances psychological well-being, and fosters adaptive coping strategies [25]. Community nurses assist patients in identifying and utilizing their inner strengths, such as resilience, hope, coping skills, and social connections, to enhance self-efficacy and resilience [26].

Unlike men, women have more biological vulnerability to depressive symptoms than men due to hormonal instability that influences low mood. Despite extensive research on the biological vulnerability of women to depressive symptoms, such as hormonal fluctuations, genetic predisposition, and neurochemical differences, less is known about how inner strengths, such as loving-kindness, mindfulness, perseverance, ethical living, and generosity, may mediate this vulnerability and serve as protective factors. Understanding this mediation effect is crucial, as it offers a holistic approach to mental health that integrates biological, psychological, and spiritual resilience mechanisms. By identifying whether and how these inner strengths mitigate depression risk, this study can contribute to targeted interventions, promoting non-pharmacological, strength-based mental health strategies for women from healthcare providers such as nurses. This study is essential for developing preventive frameworks that empower women to cultivate inner strengths as a buffer against biological stressors and depressive symptoms. Based on this rationale, the researchers hypothesize that inner strengths would significantly mediate the relationship between biological vulnerability and depressive symptoms in women. Specifically, higher engagement in these strengths will be associated with reduced depressive symptoms, regardless of biological predisposition. To examine the underlying mechanism linking biological factors and depressive symptoms, inner strength was included as a mediating variable in the structural equation model.

## 2. Materials and Methods

### 2.1. Study Design

This study employed a cross-sectional survey administered online in mainland China. The research received approval from the Research Ethics Committee of the Faculty of Medicine at Chiang Mai University. All participants provided informed consent prior to participation. The sample size (n) was calculated based on previous literature data using the formula n = z^2^ × p × (1 − p)/e^2^, where z = 1.96 for a 95% confidence level (α), p = 0.3 for the proportion (expressed as a decimal), e = 0.05 for the margin of error, and n = 1.96^2^ × 0.3 × (1 − 0.3)/0.05^2^, which approximates 323. The sample was recruited using a convenience sampling method via online platforms. We disseminated the survey invitation through social media—WeChat (Tencent Holdings Ltd., Shenzhen, China) groups and public accounts commonly used by working mothers, workplace emails, and parent associations in selected urban and regional cities. Potential participants accessed the survey link, where an introductory page included the detailed study information and consent form. Upon agreement, candidates were required to complete a preliminary eligibility screening questionnaire embedded at the beginning of the online survey, which applied all inclusion and exclusion criteria automatically. Only eligible participants were allowed to proceed to the main survey. No randomization or stratification was applied in the recruitment process. A total of 369 questionnaires were collected, among which 39 were excluded as invalid. The invalid cases were identified based on criteria, including respondents who duplicated IP addresses and had short completion times. Finally, 330 questionnaires were collected.

### 2.2. Participants

The participants consisted of working mothers from various urban cities and regional areas in China. The inclusion criteria were (1) women aged between 30–45 years, (2) at least one child aged from 6–11 who was in primary school, (3) had a full-time or part-time job, (4) women during pregnancy, premenopausal or perimenopausal period, (5) had computers or smartphones with good internet. The exclusion criteria were (1) employed but on long-term leave, (2) living in rural areas as recorded in the Chinese household registration book.

### 2.3. Procedure

Each participant received a detailed explanation of the research, including a Participant Information Sheet (PIS) and an Informed Consent Form (ICF). The questionnaire was designed exclusively for research purposes, and participants were asked to complete self-evaluation items to determine eligibility based on the inclusion and exclusion criteria. A total of 369 individuals were invited to participate through an online platform, and 330 completed questionnaires met the eligibility requirements. To acknowledge their participation, each respondent received CNY 5 via Alipay Red Envelopes. The first author downloaded the data daily to an offline database accessible to the research team. Data collection was conducted from 17 October to 21 November 2024.

### 2.4. Measurements

#### 2.4.1. Demographic and Socioeconomic Factors

Sociodemographic and socioeconomic characteristics were assessed using a self-administered questionnaire, including participants’ age, marital status, weekly working hours, educational level, and annual income.

#### 2.4.2. Outcome Inventory 21 (OI-21)

The Outcome Inventory-21 (OI-21) employs a 21-point Likert scale [27]. The self-rating process evaluated four distinct areas: depression, anxiety, somatization, and interpersonal difficulty. The ratings on this scale span from 0, which represents “never,” to 4, signifying “almost always.” The overall scores for the OI-21 can range from 0 to 84. A higher score shows the presence of a greater number of symptoms. In terms of the approach for ascertaining the prevalence, it was drawn on previous research regarding the development of depression symptoms with a cut-off score of 7 [27]. The Cronbach’s alpha of OI-21 of anxiety, depression, interpersonal difficulty, and somatization scales among all participants were 0.934, 0.934, 0.899, and 0.922, respectively.

#### 2.4.3. Inner-Strength-Based Inventory (I-SBI)

The Inner Strength-Based Inventory (I-SBI) assesses ten domains derived from the ten perfections of Buddhist doctrine [28], each representing a specific dimension of inner strength: (1) Truthfulness refers to honesty and integrity in actions and words. (2) Perseverance denotes persistent effort in achieving goals despite difficulties. (3) Wisdom is the ability to understand, reflect, and make sound judgments. (4) Generosity relates to the willingness to give, share, and support others selflessly. (5) Morality involves adherence to ethical principles and moral conduct, including refraining from killing, stealing, sexual misconduct, bad-intentioned lying, and the consumption of alcohol or addictive substances. (6) Mindfulness or meditation encompasses awareness and attentiveness to the present moment. (7) Patience and endurance represent calm tolerance in the face of adversity or delay. (8) Equanimity is the ability to maintain mental balance and composure during stress or emotional situations. (9) Determination signifies strong will and resolve in pursuing objectives. (10) Loving-kindness reflects compassion and goodwill toward oneself and others. These ten domains collectively capture the broad attributes of inner strength and may play a crucial role in promoting psychological resilience. One strength was assessed using a single multiple-choice question on a 5-point scale. Higher scores on a scale of 10 to 50 correspond to more inner strength. The Chinese version [22] of the SBI demonstrated a good reliability coefficient of 0.86 in the reliability test. In this research, the tool has Cronbach’s α = 0.878.

#### 2.4.4. Parental Stress Scale (PSS)

Authors Berry and Jones (1995) [29] developed the Parental Stress Scale, which was subsequently translated into Chinese by Cheung (2000) [30]. The positive and negative aspects of parenthood are represented among its 18 items on a five-point scale, ranging from Strongly Disagree to Strongly Agree. Parental stress increases in proportion to the score. The Chinese version of the scale exhibits satisfactory levels of validity and reliability. The alpha value of 0.89 was obtained in this group [30].

### 2.5. Statistical Analysis

Descriptive analysis techniques were employed to characterize the demographic information, mental health results, and levels of depression. These were presented in terms of the mean value and standard deviation. Sociodemographic variables were either nominal or ordinal and were summarized by presenting the relevant counts and percentages. Analysis of Variance (ANOVA) was utilized to investigate the differences existing among multiple groups. Pearson’s correlation was applied to determine the relationship between variables. Multiple regression is a method used to understand the relationship between one outcome (the dependent variable) and two or more factors that might influence it (independent variables). It helps you see how each factor affects the outcome when all the factors are considered simultaneously.

To ensure the suitability of the data for mediation analysis, multiple regression analyses were conducted across the variables. These analyses confirmed that standard error distribution and homoscedasticity assumptions were met for all regressions, supporting the validity of the mediation approach. To examine the mediation models, we first assessed the magnitude of relationships among biological factors, the inner strength score, and depression scores using zero-order correlations. To improve statistical power and model parsimony, given the modest sample size, related biological predictors (e.g., hormonal fluctuations, smoking, alcohol use, family psychiatric history, and physical diseases) were combined into a composite biological risk score. This approach accounts for the cumulative impact of biological risks on depressive symptoms, minimizes potential multicollinearity among predictors, and is aligned with theoretical models emphasizing aggregate risk. Using a composite variable allows for a more robust and interpretable mediation analysis. Mediation analysis was conducted using the PROCESS macro, a computational tool designed to estimate mediation models. It facilitates the assessment of indirect effects through bootstrapping methods, enabling researchers to examine whether another variable mediates the impact of an independent variable on a dependent variable. The data analysis was conducted using the IBM SPSS, Version 27 (IBM Corp, Armonk, NY, USA). For all the data analyzed, a *p*-value of less than 0.05 was considered significant.

## 3. Results

### 3.1. Sociodemographic and Socioeconomic Characteristics

Table 1 summarizes the sociodemographic and socioeconomic characteristics of the 330 Chinese mothers, including group comparisons and corresponding percentages. Among the participants, 83.6% were married or cohabiting, and 42.7% (n = 141) reported having two or more children. Over half of the respondents held at least a bachelor’s degree, indicating a relatively high educational attainment. The majority of employed mothers (n = 205) were engaged in full-time employment, typically working 40 h or more per week. In terms of household income, approximately 75% of the participants reported an annual income of less than CNY 150,000, indicating a predominantly lower- to middle-income population.

### 3.2. Table 2 Biological Characteristics

Table 2 summarizes the biological characteristics of the 330 participants. Core biological factors—namely, hormone fluctuations and a family history of psychiatric disorders—were observed in 17.6% of the sample. Bio-behavioral factors, defined as cigarette and alcohol use, were present in 20.3% of participants. Secondary biological factors, represented by existing physical illnesses, were reported by 22.7% of the cohort. When considering composite biological variables (i.e., the presence of any of the above factors), 37.3% of participants exhibited at least one.

### 3.3. Test Differences Between Associated Factors and Depression

The results indicated that participants who were single, divorced, widowed, or separated reported significantly higher levels of depression compared to those who were married or cohabiting. Mothers with three or more children exhibited the highest levels of depression. Lower educational attainment was also associated with increased depressive symptoms; notably, participants with less than a high school education reported the highest depression scores. Furthermore, participants working more than 54 h per week demonstrated significantly higher levels of depression, suggesting a potential association between long working hours and adverse mental health outcomes.

The findings also indicated that participants experiencing hormonal fluctuations—during pregnancy, within one year postpartum, or during perimenopause/menopause—reported significantly higher levels of depressive symptoms. Additionally, mothers who reported cigarette or alcohol use had elevated depression scores. A higher level of depressive symptoms was also observed among participants with a family history of psychiatric disorders. Furthermore, those diagnosed with physical conditions such as cardiovascular disease, hypertension, or other chronic illnesses exhibited significantly higher levels of depression (see Table 3).

### 3.4. Pearson’s Correlation Among Variables

Table 4 presents the correlation coefficients among four key variables: Composite Biological, I-SBI, OI-Depression, and PSS. Composite Biological is negatively correlated with I-SBI (−0.347 **) but positively correlated with OI-Depression (0.429 **) and PSS (0.371 **), suggesting that biological factors may contribute to depression and parental stress but inversely relate to inner strengths. I-SBI shows strong negative correlations with both OI-Depression (−0.708 **) and PSS (−0.705 **), indicating that inner strengths significantly buffer against depression and stress. OI-Depression and PSS have a high positive correlation (0.837 **), reinforcing the close link between depression and parental stress in this sample.

### 3.5. Tests of Mediation

Table 5 presents the results from a multiple regression model and a Mediation model (Model 4) that examines the relationships between biological factors, demographics, psychological variables, and depressive symptoms. In the multiple regression model, “Composite biological” significantly positively correlated with depressive symptoms (B = 1.599, *p* < 0.001), as did several demographic factors. The model accounted for 67.1% of variance (R^2^ = 0.671, F = 37.744, *p* < 0.001). In Model 4, “Composite biological” (B = 0.584, *p* < 0.001), inner strength (I-SBI, B = −0.143, *p* < 0.001), and parental stress (PSS, B = 0.192, *p* < 0.001) had significant associations. Other variables were non-significant. This model explained 75.1% of the variance (R^2^ = 0.751, F = 106.954, *p* < 0.001), illuminating the complex relationships underlying depressive symptoms.

Table 6 presents the total, direct, and indirect effects of biological factors on depressive symptoms. The results indicate a significant total effect of biological factors on depressive symptoms (β = 1.600, *p* < 0.001). The direct effect was also significant (β = 0.977, *p* < 0.001), as was the indirect effect (β = 0.623, 95% CI [0.294, 0.962]). Notably, the indirect effect accounted for 38.94% of the total effect, while the direct effect accounted for 61.06%. These findings suggest that inner strength plays a moderating role in the relationship between biological factors and depressive symptoms.

## 4. Discussion

This study demonstrates that inner strength significantly mediates the relationship between biological risk factors and depressive symptoms among Chinese working mothers. Inner strength, characterized by an individual’s capacity for emotional stability, resilience, and a positive outlook, emerged as a key protective factor against depression. Consistent with Kerstin Viglund and colleagues’ study, the inner strength served as a protective factor against negative mental health among old people [31], an inner strength acted as a protective variable for major depression, anxiety, and somatic symptoms among Chinese sales workers [22], and another study also confirmed the mediating role of inner strengths in depression [32]. The fact that inner strength seems to be a potent factor may be because it encompasses many values, including truthfulness, perseverance, wisdom, generosity, morality, mindfulness or meditation, patience and endurance, equanimity, determination, and loving kindness. These virtues, although derived from Buddhism, are universal, positive attributes that can be cultivated in non-Buddhist contexts.

Inner strengths are crucial for resilience, mental well-being, and adaptive coping. Research highlights that these traits can be developed through targeted interventions. For instance, mindfulness and meditation—core components of inner strength—are effectively promoted by practices such as Mindfulness-Based Stress Reduction (MBSR) and Mindfulness-Based Cognitive Therapy (MBCT). One 2023 Nursing Outlook study reported that nurses completing MBCT showed a 35% increase in mindfulness and a 28% decrease in burnout, underscoring the benefits for both personal well-being and professional performance [33].

Patience, endurance, and equanimity—key for managing stress and setbacks—can be strengthened through acceptance and commitment therapy (ACT). In a randomized trial, ACT participants showed notable gains in both patience (42%) and equanimity (38%) versus controls [34].

Despite these promising results, most research focuses on individual traits rather than the comprehensive I-SBI inner strength framework, underscoring the need for studies that assess all ten traits collectively. Additionally, most interventions are Western in origin, while the I-SBI is rooted in Buddhist thought, suggesting that incorporating Eastern practices could enhance cultural relevance. Nonetheless, evidence supports that these interventions are adaptable to nursing and practical for promoting well-being and professional growth, especially among working mothers. Integrating these findings into clinical education could help enhance resilience and mitigate burnout both at home and in the workplace.

It is expected that parental stress and working hours are closely associated with the occurrence of depressive symptoms. A recent study reported that 57% of parents are experiencing parenting burnout, with maternal stress being particularly pronounced. Parenting burnout is strongly linked to internal and external expectations, such as striving to meet the ideal image of a “good parent”, perceived social judgment, and the amount of time devoted to interacting with children. Excessive parenting stress is a known precipitant of maternal depression [35]. Women who work more than 55 h per week exhibit 7.3% more depressive symptoms compared to those who work 35–40 h per week, and they are more likely to feel a sense of powerlessness [36].

The findings of this study contribute to a more nuanced understanding of mental health challenges among Chinese working mothers and have practical implications for policy and healthcare practice within low-and middle-income countries (LMICs). For example, in Thailand, community-based perinatal mental health programs are integrated into primary care, enabling early identification and referral of at-risk mothers through trained nurses and village health volunteers [37]. In India, community-based interventions for maternal mental health—where lay health workers provide accessible psychosocial support—are particularly effective and preferred by mothers. Expanding such approaches, alongside further research on medication use and social support, can meaningfully improve outcomes for mothers and their children in LMICs [38]. Drawing from these models, Chinese primary care could strengthen workforce training for routine screening and reinforce community support systems. Additionally, policy improvements could focus on extending maternity leave, offering flexible work arrangements, and developing affordable, high-quality childcare services—practices endorsed in successful programs in other Asian LMICs. Such adaptations could enhance early detection and intervention for maternal mental health, as well as promote inner strength and psychological resilience among working mothers.

Regarding the prevalence of depressive symptoms, it was found that the prevalence was 38.2% for depressive symptoms, according to the self-reported screening measurement of Outcome Inventory-21. These rates are notably higher than those reported in previous research among Chinese women aged 40–60 years, where 19.5% experienced depressive symptoms [39]. One possible reason is that the previous study focused only on women, without specifically targeting the subgroup of working mothers. Another reason may be attributed to the different measurement methods used in the studies. Although clinicians should make a definitive diagnosis to find the accurate prevalence of the disorders, these prevalence rates give us concern about the mental health status of Chinese working women. It should be noted that the present study used the OI-21 for depression screening, which demonstrated high Cronbach’s alpha values for the OI-Depression subscale in this Chinese sample, indicating strong internal consistency. However, since the scale has not been formally validated in Chinese populations, questions remain about its construct and criterion validity—particularly regarding the appropriate cut-off score for depression in Chinese clinical settings. Therefore, prevalence estimates derived from this instrument should be interpreted with caution.

Growing evidence confirms that working mothers face heightened depressive symptoms due to dual professional and caregiving pressures. A Tunisian study, for example, found that 76.1% of working pregnant women exhibited depressive symptoms via the CES-D scale, reflecting increased vulnerability during the perinatal period amid competing employment and pregnancy demands [40]. Our lower overall prevalence likely stems from including but not focusing exclusively on pregnant women. Similarly, a study of urban Chinese postpartum women (within 1 year of childbirth) noted 29.42% met depression criteria, with 39.81% among commercial enterprise employees [41]—underscoring higher postpartum depression rates in working versus non-working women and linking employment stress to perinatal mental health risks. By focusing on working mothers aged 30–45, a period marked by peak career responsibilities and often ongoing parental care, research can illuminate how systemic and individual factors interact to shape psychological well-being, filling gaps in understanding of midlife mental health beyond traditional postpartum frameworks.

Overall, unlike men, women undergo natural hormonal fluctuations that can intensify depressive symptoms, particularly during critical life stages such as pregnancy, postpartum, and perimenopause. Compared to non-working women, working mothers, the risk of depressive symptoms may be further elevated due to the dual burden of professional responsibilities and childcare demands. Working mothers often experience heightened psychological strain as they navigate the competing pressures of work–life balance, excessive working hours, and parental stress. This study, however, suggests that individuals with strong inner strengths possess a protective factor against the development of depression.

### 4.1. Implications

The findings of this study provide a more comprehensive understanding of mental health challenges faced by Chinese working mothers and highlight the crucial role of public health strategies in managing depressive symptoms. Comprehensive public health measures—such as routine mental health screening, widespread mental health education, and the promotion of stress-coping and resilience-building programs—can facilitate early identification and intervention for mothers at risk. Policymakers should also prioritize structural supports, including extended maternity leave, flexible work arrangements, and increased access to high-quality childcare services, all of which can reduce psychosocial stressors and support maternal mental wellbeing. Implementing these multisectoral interventions at the community and policy levels is essential for effectively preventing and managing depression among working mothers.

### 4.2. Limitations and Recommendations for Further Research

This study has several limitations that should be acknowledged. First, the sample was restricted to urban and regional areas in mainland China. Further research should broaden the inclusion criteria to rural settings. Second, increasing the sample size and incorporating additional relevant variables would also enhance the robustness of future studies. Third, the use of a cross-sectional design precludes the establishment of causal relationships between the identified predictors and depression outcomes. Longitudinal studies and the application of random sampling methods are recommended to strengthen causal inference. Fourth, individual biological risk factors and depression symptoms were examined using only bivariate analyses. Therefore, the observed associations may not represent the independent effect of each biological risk. Future research using multivariable regression analysis is recommended to elucidate the unique contribution of each risk factor while controlling for other variables. Fifth, the prevalence data were collected through an online WeChat survey, which may limit the generalizability of the findings. Ultimately, a mixed-methods approach can address the limitations inherent in quantitative data by providing deeper contextual insights and enhancing the validity and reliability of the findings [42].

## 5. Conclusions

This study highlights the significant role of biological factors in depressive symptoms, as well as the protective effect of inner strength in mitigating the impact of biological risk factors. The findings suggest that regular training, screening, and early intervention—alongside efforts to cultivate inner strength—may help reduce the risk of depression in this population. To enhance understanding and improve intervention strategies, future studies should further explore approaches for training inner strength.

## Figures and Tables

**Table 1:** Sociodemographic and socioeconomic characteristics (n = 330).

Variables	Categories	n	%
Age	30–35	94	28.5
	36–40	141	42.7
	41–45	95	28.8
Marital status	Married/cohabiting	276	83.6
	Single	9	2.7
	Divorced/widowed/separated	45	13.6
Children number	1	189	57.3
	2	111	33.6
	≥3	30	9.1
Educational level	High school and below	81	24.6
	High vocational school	69	20.9
	Bachelor’s degree and above	180	54.5
Weekly working hours	1–20	48	14.6
	21–39	77	23.3
	40–54	147	44.5
	≥55	58	17.6
Annual income (CNY)	0–60,000	78	23.6
	61,000–10,000	88	26.7
	101,000–150,000	81	24.5
	>151,001	83	25.2

CNY = Chinese yuan (7.30 CNY = 1 U.S. Dollar).

**Table 2:** Biological characteristics (n = 330).

Variables	Categories	n	%
Core-biological	No	272	82.4
	Yes	58	17.6
Bio-behavioral	No	263	79.7
	Yes	67	20.3
Secondary biological	No	255	77.3
	Yes	75	22.7
Composite biological	0	207	62.7
	1	78	23.6
	2	31	9.4
	3	8	2.4
	4	6	1.8
Mean ± SD, min-max	0.57 ± 0.8, 0–4		
Median, interquartile range	0, 1		

**Table 3:** Depression symptoms based on the participants’ characteristics (n = 330).

Variables	OI-Depression Score (Mean ± SD)	Test Difference	*p*-Value
Age			
30–35	6.67 ± 5.7	F (2, 327) = 4.572	*p* < 0.05
36–40	4.73 ± 4.3		
41–45	5.31 ± 4.6		
Marital status			
Married/cohabiting	4.87 ± 4.5	F (2, 327) = 12.878	*p* < 0.001
Single	7.78 ± 7.3		
Divorced/widowed/separated	8.56 ± 5.4		
Children number			
1	4.03 ± 4.2	F (2, 327) = 36.693	*p* < 0.001
2	6.32 ± 5.0		
≥3	11.17 ± 3.8		
Educational level			
High school and below	9.74 ± 5.0	F (2, 327) = 55.364	*p* < 0.001
High vocational school	4.52 ± 3.8		
Bachelor’s degree and above	3.87 ± 4.1		
Weekly working hours			
1–20	5.15 ± 4.9	F (3, 326) = 16.181	*p* < 0.001
21–40	3.53 ± 4.2		
41–54	5.16 ± 4.5		
≥55	8.98 ± 5.0		
Annual income			
0–60,000	6.96 ± 5.6	F (4, 325) = 10.995	*p* < 0.001
61,000–10,000	6.74 ± 4.7		
101,000–150,000	5.36 ± 4.2		
>151,001	2.80 ± 3.9		
Hormonal fluctuations			
No	5.03 ± 4.9	F (3, 326) = 4.708	*p* < 0.01
Pregnancy	7.00 ± 4.9		
Within 1 year after delivery	8.11 ± 4.7		
Perimenopause or menopause	7.94 ± 4.4		
Smoking			
No	5.11 ± 4.7	F (1, 328) = 26.329	*p* < 0.001
Yes	10.70 ± 4.4		
Alcohol use			
No	4.96 ± 4.7	F (1, 328) = 31.888	*p* < 0.001
Yes	9.82 ± 5.0		
Family psychiatric history			
No	5.25 ± 4.8	F (1, 328) = 18.229	*p* < 0.001
Yes	11.80 ± 4.8		
Physical disease(s)			
No	4.65 ± 4.7	F (5, 324) = 8.234	*p* < 0.001
Arthritis	6.11 ± 4.4		
Cardiovascular disease	7.90 ± 5.6		
Hypertension	8.85 ± 4.7		
Diabetes	5.83 ± 4.8		
Others	9.71 ± 4.4		

OI = Outcome Inventory.

**Table 4:** Correlation coefficients among variables (n = 330).

Items	Composite Biological	I-SBI	OI-Depression	PSS
Composite biological	-			
I-SBI	−0.347 **	-		
OI-Depression	0.429 **	−0.708 **	-	
PSS	0.371 **	−0.705 **	0.837 **	-

I-SBI = inner-strength based inventory; OI = Outcome Inventory; PSS = Parental Stress Scale, ** *p* < 0.01.

**Table 5:** Mediation effect of inner strengths on the biological factors and depressive symptoms.

	β	SE	*t*	*p*-Value	LLCI	ULCI
Multiple Regression Model						
Constant	2.517	1.501	1.677	0.950		
Composite biological	1.599	0.241	6.623	<0.001		
Age	−0.310	0.280	−1.107	0.269		
Weekly working hours	0.499	0.206	2.429	0.016		
Marital status	1.069	0.533	2.006	0.046		
Educational level	−0.973	0.215	−4.536	<0.001		
Number of children	2.010	0.317	6.335	<0.001		
Annual income (CNY)	−0.432	0.175	−2.477	0.014		
R^2^	0.671 (F = 37.744, Df1 = 5, Df2 = 322, *p* < 0.001)					
Mediation model (Model 4)						
Constant	−0.310	1.568	−0.197	0.844	−3.393	2.775
Composite biological	0.584	0.171	3.406	<0.001	0.247	0.921
I-SBI	−0.143	0.024	−5.863	<0.001	−0.191	−0.095
PSS	0.192	0.015	13.208	<0.001	−0.163	0.220
Age	−0.104	0.189	−0.547	0.585	−0.476	0.269
Weekly working hours	0.241	0.140	1.727	0.085	−0.034	0.516
Marital status	0.668	0.362	1.843	0.066	−0.045	1.380
Educational level	−0.219	0.152	−1.446	0.149	−0.518	0.079
Number of children	−0.480	0.251	−1.916	0.056	−0.973	0.013
Annual Income (CNY)	0.234	0.124	1.881	0.061	−0.011	0.479
R^2^	0.751 (F = 106.954, Df1 = 9, Df2= 320, *p* < 0.001)					

β = standardized coefficient, SE = standard error, LLCI = lower-level confidence interval, ULCI = upper-level confidence interval, Df = degree of freedom, I-SBI = inner-strength based inventory, PSS = Parental Stress Scale, and CNY = Chinese yuan (7.30 CNY = 1 U.S. Dollar).

**Table 6:** Direct and indirect effects of biological factors on depressive symptoms.

	β	S.E.	LLCI	ULCI	*p*-Value	Effective Size
Total effect	1.600	0.242	1.124	2.074	0.000	
Direct effect	0.977	0.210	0.564	1.389	0.000	61.06%
Indirect effect	0.623	0.170	0.294	0.962		38.94%

## Data Availability

According to the policy implemented during this study, the ethics committee does not permit the authors to share the data with other entities. The data sets used and/or analyzed for the current study are available from the corresponding author upon reasonable request.

## Public Involvement Statement

There was no public involvement in any aspect of this research.

## Guidelines and Standards Statement

This manuscript was drafted in accordance with the Strengthening the Reporting of Observational Studies in Epidemiology (STROBE) guidelines for cross-sectional studies.

## Abbreviations

The following abbreviations are used in this manuscript:

OI-21	Outcome Inventory 21
I-SBI	Inner-Strength-Based Inventory
PSS	Parental Stress Scale
ANOVA	Analysis of Variance
